# Privacy-Preserving Generative Deep Neural Networks Support Clinical Data Sharing

**DOI:** 10.1161/CIRCOUTCOMES.118.005122

**Published:** 2019-07-09

**Authors:** Brett K. Beaulieu-Jones, Zhiwei Steven Wu, Chris Williams, Ran Lee, Sanjeev P. Bhavnani, James Brian Byrd, Casey S. Greene

**Affiliations:** 1 Genomics and Computational Biology Graduate Group, Perelman School of Medicine, University of Pennsylvania, Philadelphia. (B.K.B.-J.); 2 Department of Systems Pharmacology and Translational Therapeutics, Perelman School of Medicine, University of Pennsylvania, Philadelphia. (C.W., C.S.G.); 3 Computer Science and Electrical Engineering Department, University of Minnesota, Minneapolis (Z.S.W.).; 4 Division of Cardiovascular Medicine, Department of Medicine, University of Michigan Medical School, Ann Arbor (R.L., J.B.B.).; 5 Scripps Clinic and Research Foundation, San Diego, CA (S.P.B.).

**Keywords:** blood pressure, deep learning, machine learning, privacy, propensity score

## Abstract

**Background:**

Data sharing accelerates scientific progress but sharing individual-level data while preserving patient privacy presents a barrier.

**Methods and Results:**

Using pairs of deep neural networks, we generated simulated, synthetic participants that closely resemble participants of the SPRINT trial (Systolic Blood Pressure Trial). We showed that such paired networks can be trained with differential privacy, a formal privacy framework that limits the likelihood that queries of the synthetic participants’ data could identify a real a participant in the trial. Machine learning predictors built on the synthetic population generalize to the original data set. This finding suggests that the synthetic data can be shared with others, enabling them to perform hypothesis-generating analyses as though they had the original trial data.

**Conclusions:**

Deep neural networks that generate synthetic participants facilitate secondary analyses and reproducible investigation of clinical data sets by enhancing data sharing while preserving participant privacy.

## Abstract 2

Supplemental Digital Content is available in the text.

Sharing individual-level data from clinical studies remains challenging. The status quo often requires scientists to establish a formal collaboration and execute extensive data usage agreements before sharing such data. These requirements slow or even prevent data sharing between researchers in all but the closest collaborations. Individual-level data is critical for certain secondary data analyses (eg, propensity score matching techniques) and subgroup analyses.^1^

Even for efforts specifically designed to highlight the value of sharing data, investigators have been required to execute data use agreements. The *New England Journal of Medicine* recently held the SPRINT (Systolic Blood Pressure Trial) Data Analysis Challenge to examine possible benefits of clinical trial data sharing.^2,3^ The SPRINT clinical trial examined the efficacy of intensive lowering of systolic blood pressure (<120 mm Hg) compared with treatment to a standard systolic blood pressure goal (<140 mm Hg). Intensive blood pressure lowering resulted in fewer cardiovascular events, and the trial was stopped early for benefit. Reanalysis of the challenge data led to the development of personalized treatment scores^4^ and decision support systems,^5^ in addition to a more specific analysis of blood pressure management in participants with chronic kidney disease.^6^ The goal of these agreements is to maintain participant privacy by prohibiting reidentification or unauthorized disclosure.

We sought to find a way to share data for initial and exploratory analyzes that does not require this data use agreement process. To do this, we developed a technical solution for generating synthetic participants that were similar enough to the original trial data that both standard statistical and machine learning analyses yield effectively the same answers. Other methods aimed at performing this task generally fall into 2 groups: (1) sampling methods with a quantifiable privacy risk,^7^ or (2) generative adversarial networks (GANs),^8^ which are neural networks that can generate realistic data from complex distributions. In a GAN, 2 neural networks are trained against each other: one is trained to discriminate between real and synthetic data (the discriminator), and the other is trained to generate synthetic data (the generator). GANs have become a class of widely used machine learning methods and have recently been used in biology and medicine^9^ and have been used to generate biomedical data.^10,11^ However, using traditional GANs for this task provides no guarantee on what the synthetic data reveal about true participants. It is possible that the generator neural network could learn to create synthetic data that reveals actual participant data. One way to avoid this scenario, in which a participant’s sensitive information could be revealed, is to use differential privacy. Differential privacy allows the release of aggregate statistical information about a population without compromising the privacy of any individual in the population. In particular, differential privacy promises to protect individual subjects from any additional harm that they might face due to their data being in a study that they would not have faced had they opted out of the study.

As a concrete example, suppose that a 40-year-old man John holds a health insurance policy. His premium is set at $3000 based on average healthcare reimbursements for his age and gender. A portion of his premium is due to the possibility of a stroke, say $9 if men his age have a 0.03% chance of a stroke costing $30 000. John is considering whether or not to participate in a medical study, but since he has poorly controlled hypertension, he is concerned the study will reveal he is more likely to suffer a stroke than an average male. If we suppose John opts out of the research study and the study reveals that those with poorly controlled hypertension are 3× more likely to suffer a stroke. Despite not participating in the study, John’s insurance company may update John’s premium to $3027 ($9 current expected cost of stroke×3 times more likely=$27).

Now suppose John opts into the study and the researchers conclude he is 3× more likely to suffer a stroke. During the study, the researchers found John has an additional risk factor specific to him which increases his risk of stroke within the next year to at least 20%. Would his premium increase substantially due to his participation? Differential privacy ensures that would not happen. In particular, if the researchers use a value of ε=1, then the insurance company’s estimate of the probability that John will suffer a stroke in the next year can increase from 0.09% to at most 0.09% (1+1)=0.18%. Thus, John’s insurance premium can increase from $3000 to, at most, $3054. In other words, John’s cost of participating in the study, in terms of an additional increase in her insurance premium, is at most $27.

Nissim et al^12^ provide a particularly useful primer on understanding differential privacy for a nontechnical audience as well as how to assess values for specific privacy parameters. Differential privacy has been adopted by the US Census Bureau for the 2020 US Census. The Census Bureau also provides guidance on choosing an appropriate privacy loss.^13,14^ A general background of differential privacy can be found in Dwork and Roth,^15^ and Abadi et al^16^ introduced differential privacy for deep learning.

In this study, we introduce differential privacy to the GAN framework and evaluate the extent to which differentially private GANs could generate biomedical data that can be shared for valid reanalysis while controlling participant privacy risks. We achieve differential privacy by limiting the maximum influence of any single participant during training and then adding a small amount of random noise.^16^ More detailed technical explanations of our usage of differential privacy can be found in the Methods in the Data Supplement. We evaluated usefulness by (1) comparing variable distributions between the real and simulated data, (2) comparing the correlation structure between variables in the real and simulated data, (3) a blinded evaluation of individual-level data by 3 clinicians, and (4) comparing predictors constructed on real versus simulated data. The method generates realistic data by each of these evaluations.

## Methods

We used a type of GAN known as an auxiliary classifier generative adversarial network (AC-GAN)^17^ to simulate participants based on the population of SPRINT clinical trial. We included all participants with measurements for the first 12 SPRINT visits (n=6502), dividing them into a training set (n=6000) and a test set (n=502). To evaluate the effect of applying differential privacy during the generation of synthetic participant data, we trained 2 AC-GANs using the training set: a traditional, standard AC-GAN (results termed nonprivate throughout the remainder of this article) and an AC-GAN trained under differential privacy (results termed private). We used both GANs to simulate data that we then compared to the real SPRINT data by visualizing participant blood pressure trajectories, analyzing variable correlation structure and evaluating whether predictive models trained on synthetic data achieve similar performance to models trained on real data. Three clinicians attempted to predict whether participants were real or synthetic and whether they were in the standard or intensive treatment group.

### AC-GAN for SPRINT Clinical Trial Data

An AC-GAN (Figure IA in the Data Supplement) is made up of 2 neural networks competing with each other. Details about the neural network architectures are available in the Methods in the Data Supplement. We trained the generator (G) to take in a specified treatment arm (standard/intensive) and random noise and generate new participants that can fool the discriminator (D). The generator takes in specified treatment arm to generate participants that belong to the specified arm. This labeling and additional task is the difference between an AC-GAN and a standard GAN. The generator simulated a systolic blood pressure, diastolic blood pressure, and a number of medications for each synthetic patient for each of 12 SPRINT study visits. We trained the discriminator to differentiate real and simulated data from a data set containing both groups. We repeated this process until the generator created synthetic participants that were difficult to discriminate from real ones (ie, the accuracy of the discriminator could not improve much above ≈50%).

### Training With Differential Privacy

To limit the possibility that a participant’s trial involvement could be identified, we need to limit the influence any single study participant has on the neural network training of the discriminator, the only part of the AC-GAN that accesses real data. Neural networks are trained using gradient descent, by adjusting weights according to the gradient of a loss function. Nontechnically, this means taking a series of steps that provide a more accurate output. To incorporate differential privacy, we limit the maximum distance of any of these steps and then add a small amount of random noise. A detailed explanation of the processes is given in Methods in the Data Supplement and Abadi et al.^16^

### SPRINT Clinical Trial Data

SPRINT was a randomized, single-blind treatment trial that divided hypertensive participants to either intensive treatment with a systolic blood pressure target of <120 mm Hg or standard treatment with a systolic blood pressure target of <140 mm Hg. The trial included a total of 9361 participants. We included 6502 participants who had blood pressure measurements for each of the first 12 measurements (RZ, 1M, 2M, 3M, 6M, 9M, 12M, 15M, 18M, 21M, 24M, and 27M). We included measurements for systolic blood pressure, diastolic blood pressure, and the count of medications prescribed to each participant, for a total of 3 parameters assessed at 12-time points.

### Clinician Evaluation

Three physicians made a blinded real or synthetic judgment for each of 100 figures showing systolic blood pressure, diastolic blood pressure, and number of medications at each of 12 visits. These cardiologists classified how realistic the patients looked (from 1 to 10, where 10 is most realistic) and whether the patients had been randomized to SPRINT’s standard or intensive treatment arm. Before reviewing the figures and regularly during the review of figures, the clinicians reviewed the published SPRINT protocol to help contextualize the data. We performed a Mann-Whitney *U* test to evaluate whether the real or synthetic samples received significantly different scores and compared the accuracy of the treatment arm classifications.

### Transfer Learning Task in SPRINT Trial

Each of the 6502 participants in our analytical data set was labeled by treatment arm. We evaluated machine learning methods (logistic regression, support vector machines, and random forests from the scikit-learn^18^ package) by their ability to predict a participant’s treatment arm. This was done by splitting the 6502 participants into a training set of 6000 participants (referred to as real in this article) and a test set of 502 participants. We then trained 2 AC-GANs using the 6000 participant training set, (1) an AC-GAN model trained without differential privacy (referred to as nonprivate) and (2) an AC-GAN trained with differential privacy (referred to as private). Each classifier was then trained on 3 data sets, (1) the real training data set, (2) synthetic participants generated by the nonprivate AC-GAN, and (3) synthetic participants generated by the private AC-GAN. Each classifier was then evaluated on the same, real test set of participants. This allows for a comparison of classification performance (measured by area under the receiver-operator characteristic curve) between models trained on the real data, synthetic data, and private synthetic data. We evaluated both accuracy as well as the correlation between important features (random forest) and model coefficients (logistic regression and support vector machine).

### Predicting Heart Failure in the Medical Information Mart for Intensive Care Critical Care Database

We generated synthetic patients for the purpose of predicting heart failure. MIMIC is a database of 46 297 deidentified electronic health records for critical care patients at Beth Israel. We defined patients who suffered from heart failure as any patient in MIMIC diagnosed with an *International Classification of Diseases, Ninth Revision* code included in the Veterans Affairs’ Chronic Heart Failure Quality Enhancement Research Initiative’s guidelines: (402.01, 402.11, 402.91, 404.01, 404.03, 404.11, 404.13, 404.91, 404.93, 428, 281.1, 428.20, 428.21, 428.22, 428.23, 428.30, 428.31, 428.32, 428.33, 428.40, 428.41, 428.42, 428.43, and 428.9). We performed complete case analysis for patients with at least 5 measurements for mean arterial blood pressure, arterial systolic and diastolic blood pressures, beats per minute, respiration rate, peripheral capillary oxygen saturation (Spo_2_), mean noninvasive blood pressure, and mean systolic and diastolic blood pressures. For patients with >5 measurements for these values, the first 5 were used. This yielded 8260 total patients and 2110 cases of heart failure. We included the first 7500 patients in the training set and the remaining 760 in a validation set. The training and transfer learning procedures matched SPRINT protocol.

## Results

**Figure 1.**
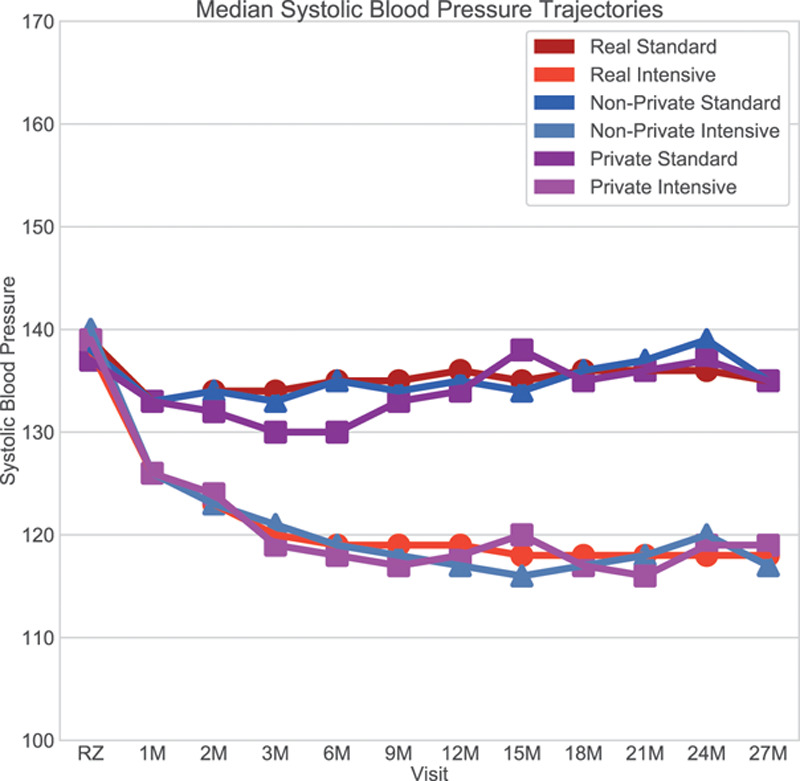
**Median systolic blood pressure trajectories from initial visit to 27 mo**.

**Figure 2.**
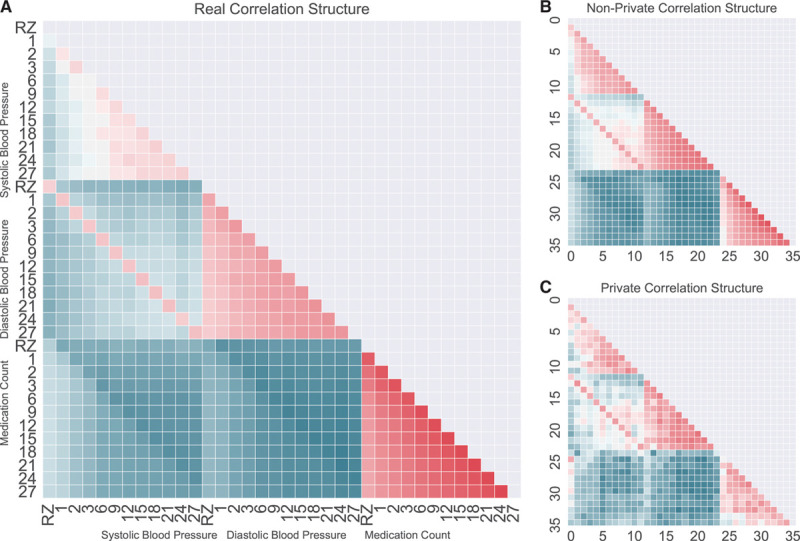
**Pairwise Pearson correlation between columns. A**, Original and real data, (**B**) nonprivate and auxiliary classifier generative adversarial network (AC-GAN) simulated data, and (**C**) differentially private and AC-GAN simulated data (RZ, randomization visit; 1M, 1-mo visit; 2M, 2-mo visit; 3M, 3-mo visit; 6M, 6-mo visit; 9M, 9-mo visit; 12M, 12-mo visit; 15M, 15-mo visit; 18M, 18-mo visit; 21M, 21-mo visit; 24M, 24-mo visit; and 27M, 27-mo visit).

We trained a differentially private AC-GAN to generate 5000 synthetic participants that resemble the real trial participants (Figure 1). Because the AC-GAN was trained under differential privacy, we could either release the model or generate as many patients as desired without an additional impact on training as differential privacy is robust to post-processing.^15^ We compare the median systolic blood pressures over time (Figure 2) of 3 groups, (1) real participants (real), (2) simulated participants via a nonprivate AC-GAN (nonprivate), and (3) simulated participants via the differentially private AC-GAN (private). The nonprivate participants generated at the end of training appear similar to the real participants. The private participants have wider variability because of the noise added during training (Figure 1A).

The Table compares how close statistics calculated between the 3 groups were, as well as a comparison of treatment decisions between the real and synthetic participants. In particular, we examined the proportion of times an additional medication was added when a participant was above the target systolic blood pressure goal for their treatment arm (120 mm Hg for intensive and 140 mm Hg for standard). For this task, the private synthetic participants closely reflected the original trial (15.51% versus 15.14%). This demonstrates the potential to meaningfully ask questions using synthetic data before acquiring and confirming a putative relationship in the real data.

**Table.**
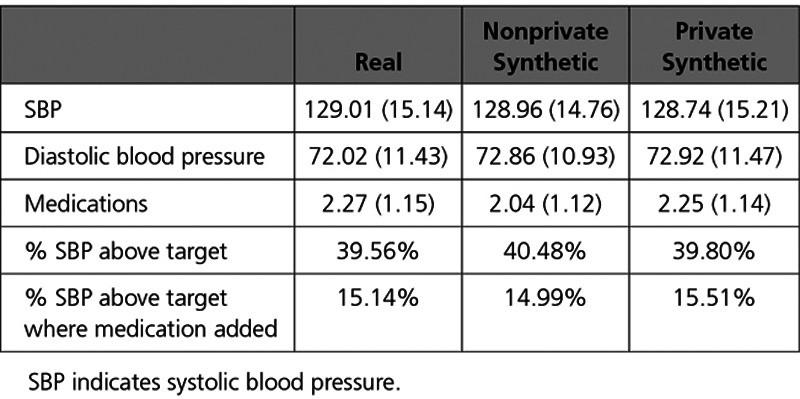
Summary Statistic Comparison Between Real, Nonprivate Synthetic, and Private Synthetic Participants, Mean (SD)

As another method of determining whether the resulting synthetic data are similar to the real data, we measured the correlation between each study visit’s systolic blood pressure, diastolic blood pressure, and medication count. We performed this analysis within SPRINT data set (real correlation structure) and within the data sets generated by the GAN without and the GAN with differential privacy (nonprivate correlation structure and private correlation structure, respectively). The Pearson correlation structure of the real SPRINT data (Figure 2A) was closely reflected by the correlation structure of the nonprivate generated data (Figure 2B). Of note was initial positive correlation between the number of medications a participant was taking and the early systolic blood pressures, but this correlation decreased as time goes on. The Pearson correlation structure (ie, the values below the diagonal in Figure 2A and 2B) for the real SPRINT data (ie, the training data) and the nonprivate data were highly correlated (Spearman correlation=0.9645; *P* value <0.0001). Addition of differential privacy during the synthetic data generation process (ie, the private data set) generated data generally reflecting these trends, but with an increased level of noise (Figure 2C). The correlation matrices between the real SPRINT data and the private generated data were only slightly less correlated (Spearman correlation=0.9185; *P* value <0.0001). The noisy training process of the private discriminator places an upper bound on its ability to fit the distribution of data. Increased sample sizes (such as in EHRs or other real-world data sources) would help to clarify this distribution and because larger sample sizes cause less privacy loss, less noise would need to be added to achieve an acceptable privacy budget.

### Human Comparison of Real Versus Synthetic Participants

**Figure 3.**
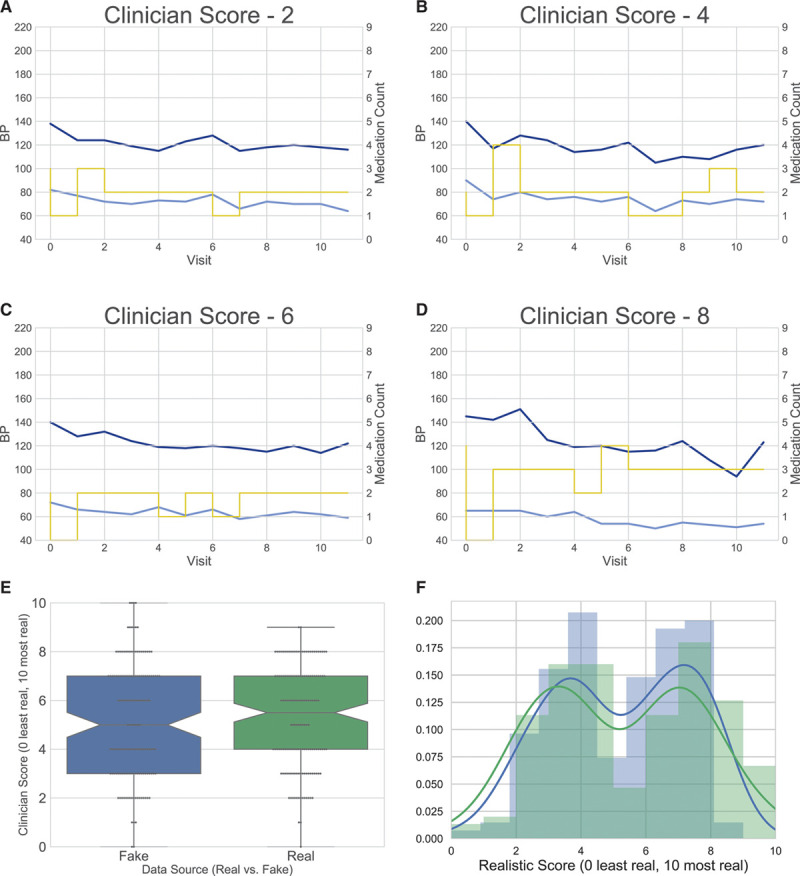
**Clinician evaluation of synthetic data. A**, Synthetic participant scored a 2 by clinician expert. **B**, Synthetic participant scored a 4 by clinician expert. **C**, Synthetic participant scored a 6 by clinician expert. **D**, Synthetic participant scored an 8 by clinician expert. **E**, Comparison of scores between real and synthetic participant (dotted red lines indicate means). **F**, Distribution of scores between real (blue) and synthetic (green) patients. BP indicates blood pressure.

To ensure similarity between the synthetic and real SPRINT data persists during rigorous inspection at more granular scale, we asked 3 clinicians to judge whether individual participant data were real SPRINT data or synthetic data. These 3 physicians, experienced in the treatment of hypertension and familiar with SPRINT trial, were each asked to determine in a blinded fashion whether 100 participants (50 real and 50 synthetic) looked real. The clinicians looked for data inconsistent with SPRINT protocol or that otherwise appeared anomalous. For example, the clinicians were alert for instances in which the systolic blood pressure was <100 mm Hg, but the participant was prescribed an additional medication. The clinicians classified each record on a 0 to 10 realism scale (10 was the most realistic), as well as whether the data correspond to standard or intensive treatment for 100 participants each (Figure 3A through 3D). The mean realism score for the synthetic patients (N=150) was 5.18 and the mean score for the real patients 5.26 (N=150; Figure 3E). We performed a Mann-Whitney *U* test to evaluate whether the scores were drawn from significantly different distributions and found a *P* value of 0.333. The clinicians correctly classified 76.7% of the real SPRINT participants and 82.7% of the synthetic participants as the standard or intensive group. During this process, without prior instruction, the clinicians followed a couple of interesting patterns which were confirmed via interview: (1) they tried to avoid choosing 5 as it would not provide any signal as to whether they thought the example was real or synthetic and (2) they generally did not feel confident enough to select extreme scores on either side. These behaviors can be seen in the resulting bimodal distribution.

### Machine Learning Models Trained on Simulated Participants Are Accurate for Real Participants

**Figure 4.**
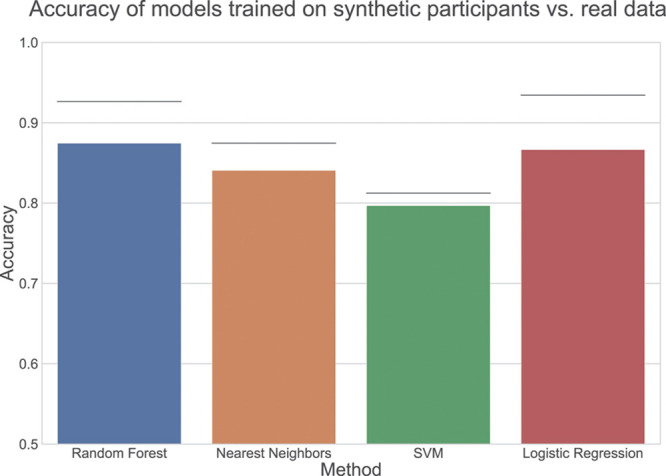
**Accuracy of models trained on synthetic participants vs real data.** Line indicates performance on real data, which on average should provide the best possible performance; bar indicates performance of classifier trained on private synthetic participants; bottom of chart indicates random performance.

Clinician review, visualizations of participant distributions, and variable correlations showed that synthetic participants appeared similar to real participants. Next, we sought to determine whether or not subsequent data analyses using synthetic data matched that of the real data. To do this, we trained machine learning classifiers using 4 methods (logistic regression, random forests, support vector machines, and nearest neighbors) to distinguish treatment arms on 3 different sources of data: real participants, synthetic participants generated by the nonprivate model, and synthetic participants generated by the private model. We compared performance of these classifiers on a separate holdout test set of 502 real participants that were not included in the training process (Figure 4). A drop in performance was expected because adding noise to maintain privacy reduces signal. If desired, training a nonprivate model could provide an approximate upper bound for expected performance.

We also sought to determine the extent to which the classifiers trained on real versus synthetic data were relying on the same features to make their predictions (Figure VI in the Data Supplement). We found that there was significant correlation between the importance scores (random forest) and coefficients (support vector machine and logistic regression) for the models trained on real versus synthetic data (Table I in the Data Supplement). In addition, it is important to note that the models achieved their performance while relying on >10 features at relatively even levels (Figure VI in the Data Supplement), demonstrating the ability to capture multivariate correlations. Finally, we tested the correlation between the first cross-validation fold with each other fold within the real data to set an upper bound of expected correlation (Figure VII in the Data Supplement).

### Privacy Analysis

We evaluate privacy based on the (ε, δ) formulation of differential privacy.^15^ This formal definition of differential privacy has 2 parameters. The parameter ε measures the maximum data set shift that could be observed by adding or removing a single participant (termed privacy loss). The second parameter, δ, is the upper bound of the probability that the privacy loss exceeds ε. Put in other words, ε represents the maximum privacy loss where there is no privacy breach, and δ represents the probability of a privacy breach. We frame the problem in this way because it is impossible to anticipate all future methods of attack. For further details refer to the Methods in the Data Supplement.

**Figure 5.**
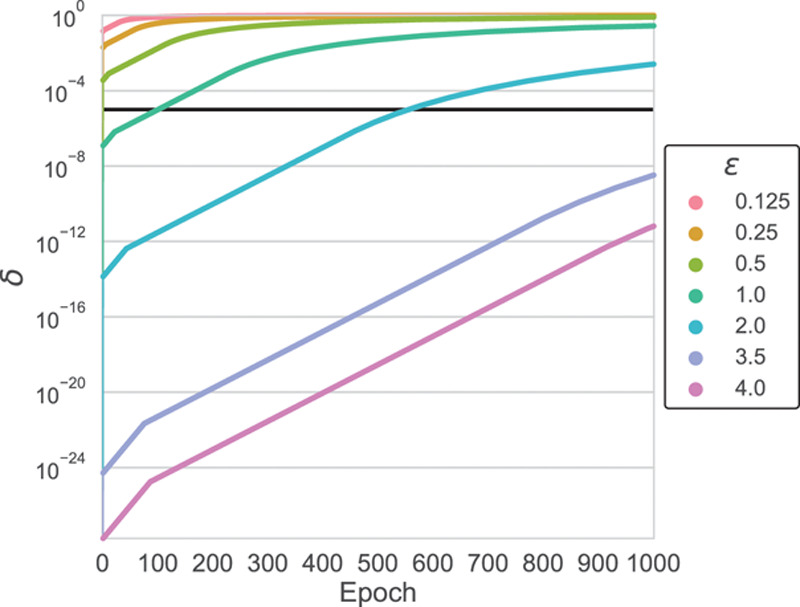
**The value of delta as a function of epoch for different ε values.** An ε value of 3.5 allows for 1000 epochs of training and δ<10^−5^.

Therefore, it is important to choose values for ε and δ that are satisfactory to the specific use case and correspond to the consequences of a privacy breach. The values of (ε, δ) increase as the algorithm (the discriminator from the AC-GAN) accesses the private data. In our experiment, our private AC-GAN algorithm is able to generate useful synthetic data with ε=3.5 and δ<10^−5^ (Figure 5). The upper bound of the epoch selection task (Methods in the Data Supplement) used (0.05, 0) per each model included for a total of (0.5, 0) differential privacy. This established a modest, single-digit ε privacy budget of (4, 10^−5^) that is on par or lower than other methods using deep learning with differential privacy.

### Predicting Heart Failure in the MIMIC Critical Care Database

**Figure 6.**
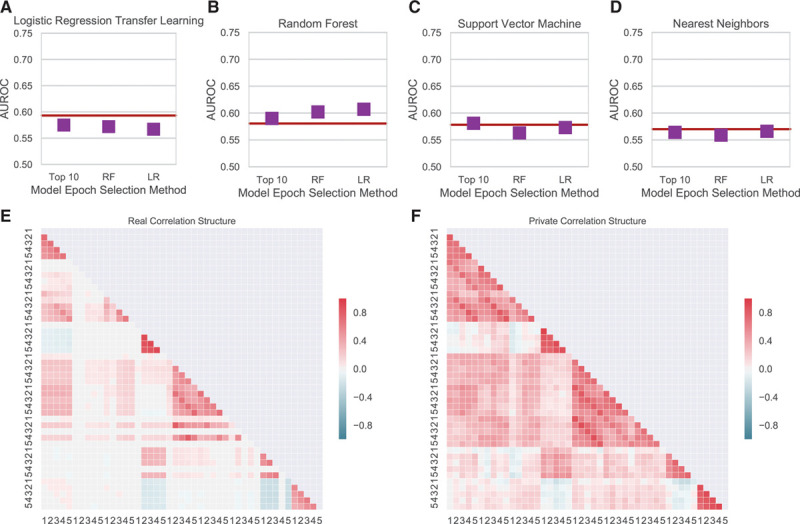
**Machine learning and statistical evaluation of synthetic data. A–D**, Performance on transfer learning task by source of training data for each machine learning method. **E**, Pairwise Pearson correlation between columns for the original and real data. **F**, Pairwise Pearson correlation between columns for the private synthetic data. AUROC indicates area under the receiver operator characteristic; LR, logistic regression; RF, random forest.

We applied the method to the MIMIC Critical Care Database^19^ to demonstrate its generality. We tested whether our approach could be applied in a second data set by predicting heart failure from the first 5 measurements for 9 vital sign measurements in 7222 patients. The vital sign measurements included: mean arterial blood pressure, arterial systolic and diastolic blood pressures, beats per minute, respiration rate, peripheral capillary oxygen saturation (Spo_2_), mean noninvasive blood pressure, and mean systolic and diastolic blood pressures. Performance on privately generated synthetic patients was on par with performance models trained on real patients (Figure 6A through 6D). As in SPRINT data, the coefficients for logistic regression and the support vector machine as well as the feature importances were significantly correlated between real and synthetic data (Table II in the Data Supplement).

## Discussion

Deep GANs and differential privacy offer a technical solution to the challenge of sharing biomedical data to facilitate exploratory analyses. Our approach, which uses deep neural networks for data simulation, can generate synthetic data to be distributed and used for secondary analysis. We perform training with a differential privacy framework that limits study participants’ privacy risk. We apply this approach to data from SPRINT clinical trial due to its recent use for a data reanalysis challenge.

We introduce an approach that samples from multiple epochs to improve performance while maintaining privacy. However, this is an early stage work and several challenges remain. Deep learning models have many training parameters and require substantial sample sizes, which can hamper this method’s use for small clinical trials or targeted studies. In this study, we demonstrated the ability to use differentially private AC-GANs on relatively low-dimensional time series data sets. We applied our method to time series as we believe this provided a better test than simple point in time data because there would be time-based correlation structures. We expect this approach to be most well suited to sharing specific variables from clinical trials to enable wide sharing of data with similar properties to the actual data. We do not intend the method to be applied to generate high-dimensional genetic data from whole genome sequences or other such features. Application to that problem would require the selection of a subset of variants of interest or substantial additional methodological work.

Another fruitful area of use may be large electronic health records systems, where the ability to share synthetic data may aid methods development and the initial discovery of predictive models. Similarly, financial institutions or other organizations that use outside contractors or consultants to develop risk models might choose to share generated data instead of actual client data. In very large data sets, there is evidence that differential privacy may even prevent overfitting to reduce the error of subsequent predictions.

Though our approach provides a general framing, the precise neural network architecture may need to be tuned for specific use cases. Data with multiple types presents a challenge. EHRs contain binary, categorical, ordinal, and continuous data. Neural networks require these types to be encoded and normalized, a process that can reduce signal and increase the dimensionality of data. New neural networks have been designed to deal more effectively with discrete data.^20,21^ Researchers will need to incorporate these techniques and develop new methods for mixed types if their use case requires it.

Due to the fluid nature of security and best practices, it is important to choose a method which is mathematically provable and ensures that any outputs are robust to post-processing. Differential privacy satisfies both needs and is thus being relied on in the upcoming 2020 United States Census.^22^ It is imperative to remember that to receive the guarantees of differential privacy a proper implementation is required. We think testing frameworks to ensure accurate implementations are a promising direction for future work, particularly in domains with highly sensitive data like health care.

The practice of generating data under differential privacy with deep neural networks offers a technical solution for those who wish to share data to the challenge of patient privacy. This technical work complements ongoing efforts to change the data sharing culture of clinical research.

## Acknowledgments

We thank Jason H. Moore (University of Pennsylvania), Aaron Roth (University of Pennsylvania), Gregory Way (University of Pennsylvania), Yoseph Barash (University of Pennsylvania), Anupama Jha (University of Pennsylvania), and Blanca Himes (University of Pennsylvania) for their helpful discussions. This article was prepared using SPRINT_POP Research Materials obtained from the National Heart, Lung, and Blood Institute (NHLBI) Biologic Specimen and Data Repository Information Coordinating Center and does not necessarily reflect the opinions or views of the SPRINT_POP or the NHLBI. We thank the participants of the SPRINT trial (Systolic Blood Pressure Trial) and the entire SPRINT Research Group. Drs Beaulieu-Jones and Greene conceived the study. Dr Beaulieu-Jones and C. Williams performed initial analyses. Drs Beaulieu-Jones and Wu designed and validated the privacy approach. Dr Byrd performed a blinded review of records. Drs Beaulieu-Jones, Greene, and Wu wrote the article and all authors revised and approved the final article. The authors have no competing interests to disclose. All data used in this article are available via the NHLBI (https://biolincc.nhlbi.nih.gov/studies/sprint_pop/), the source code is available via GitHub^23^ (https://github.com/greenelab/SPRINT_gan), and an archived version is available via Figshare^24^ (doi: 10.6084/m9.figshare.5165737).

## Sources of Funding

This study was supported by the Gordon and Betty Moore Foundation under a Data-Driven Discovery Investigator Award to Dr Greene (GBMF 4552). Dr Beaulieu-Jones was supported by a Commonwealth Universal Research Enhancement Program grant from the Pennsylvania Department of Health and by US National Institutes of Health grants AI116794, LM010098, and T15LM007092. Dr Wu is funded in part by a subcontract on the Defense Advanced Research Projects Agency Brandeis project and a grant from the Sloan Foundation. Dr Byrd is funded by US National Institutes of Health grant K23-HL128909.

## Disclosures

None.

## Supplementary Material
