# Supplementary material for: Privacy-Preserving Generative Deep Neural Networks Support Clinical Data Sharing
Source: Circ Cardiovasc Qual Outcomes. 2019 Jul 9;12(7):e005122. doi: 10.1161/CIRCOUTCOMES.118.005122 (PMC7041894; doi:10.1161/CIRCOUTCOMES.118.005122)
Supplement: Supplementary file 1 [file hcq-12-e005122-s001.pdf]

SUPPLEMENTAL MATERIAL

Supplementary Online Methods:

We developed an approach to train auxiliary classifier generative adversarial networks (AC-GANs) in a differentially private manner to enable privacy preserving data sharing. Generative adversarial networks offer the ability to simulate realistic-looking data that closely matches the distribution of the source data.

AC-GANs add the ability to generate labeled samples. By training AC-GANs under the differential privacy framework we generated realistic samples that can be used for initial analysis while guaranteeing a specified level of participant privacy.

The source code for all analyses is available under a permissive open source license in our repository [1]. In addition, continuous analysis [2] was used to re-run all analyses, to generate docker images matching the environment of the original analysis, and to track intermediate results and logs. These artifacts are freely available [3].

Background

A pair of recent preprints have reported generation of synthetic individual participant data via neural networks [4, 5]. For example, Esteban et al., generated synthetic patient data and showed that a neural network could not distinguish between the synthetic data and real data. However, it is not enough to simply build synthetic participants. Numerous linkage and membership inference attacks on both biomedical datasets [6-13] and from machine learning models [14-16] have demonstrated the ability to re-identify participants or reveal participation in a study.

To provide a formal privacy guarantee, we built GANs to generate realistic synthetic individual participant data with mathematical properties like those of the original participants' data, adding the extra protection of differential privacy [17]. Differential privacy protects against common privacy attacks including membership inference, homogeneity and background knowledge attacks. Informally, differential privacy requires that no single study participant has a significant influence on the information released by the algorithm (see Materials and Methods for a formal definition). Despite being a stringent notion, differential privacy allows us to generate new plausible individuals while revealing almost nothing about any single study participant. Within the biomedical domain, Simmons and Berger developed a method using differential privacy to enable privacy preserving genome-wide association studies [18]. Recently, methods have also been developed to train deep neural networks under differential privacy with formal assurances about privacy risks [19, 20]. In the context of a GAN, the discriminator is the only component that accesses the real, private, data. By training the discriminator under differential privacy, we can produce a differentially private GAN framework.

Auxiliary Classifier Generative Adversarial Network

We implemented the AC-GAN as described in Odena et al. [21] using Keras [22] to simulate systolic and diastolic blood pressures as well as the number of hypertension medications prescribed. Results shown use a latent vector of dimension 100, a learning rate of 0.0002, and a batch size of 1 trained for 500 epochs. To conform with the privacy claims laid out in Abadi et al. [19], gradients must be clipped per example, in our implementation this requires the batch size to be 1. To handle edge cases and mimic the sensitivity of the real data measurements, we

take the floor of zero or the simulated value and convert all values to integers. Full implementation details can be seen in the GitHub repository [1].

We chose convolutional layers because of the structure imposed by sequential measurements made during the clinical trial. The features were ordered according to timing, so local structure was tied to temporality. We used deep convolutional neural networks for both the generator and discriminator (Supp. Fig. 1B, 1C).

Differential Privacy

Differential privacy is a stability property for algorithms, specifically for randomized algorithms [23]. Informally, it requires that the change of any single data point in the data set has little influence on the output distribution by the algorithm. To formally define differential privacy, let us consider X as the set of all possible data records in our domain. A dataset is a collection of n data records from X. A pair of datasets D and D' are neighboring if they differ by at most one data record. In the following, we will write R to denote the output range of the algorithm, which in our case correspond to the set of generative models.

Definition 1 [Differential Privacy [17]]: Let $\varepsilon, \delta > 0$. An algorithm $A: X^n \rightarrow R$ satisfies (ε, δ)-differential privacy if for any pair of neighboring datasets D, D', and any event $S \subseteq R$, the following holds

$$\Pr[A(D) \in S] \leq \Pr[A(D') \in S] \exp(\varepsilon) + \delta,$$

where the probability is taken over the randomness of the algorithm.

A crucial property of differential privacy is its resilience to post-processing --- any data independent post-processing procedure on the output by a private algorithm remains private.

More formally:

Lemma [Resilience to Post-Processing]: Let algorithm A: $X^n \rightarrow R$ be an (ε, δ)-differentially private algorithm. Let A' : R → R' be a "post-processing" procedure. Then their composition of running A over the dataset D, and then running A' over the output A(D) also satisfies (ε, δ)-differential privacy.

Robustness to post-processing is critical to our application because it means that all downstream uses of the data are also (ε, δ)-differentially private. Therefore, by making the discriminator, the only part of the system that accesses the real data, differentially private, the rest of the system is also differentially private.

Determination of Privacy Budget

The privacy budget is determined a priori and takes the form of (ε, δ). In non-technical terms, ε, represents the amount the results of an analysis can change due to a single study participant and δ represents the likelihood that δ is exceeded. Therefore, the problem becomes an optimization where the goal is to minimize both (ε, δ) while still generating the same conclusions from the analysis that is performed. Within deep learning, the current standard is to achieve $\varepsilon < 10$ and $\delta < 10^{-4}$ in accordance with Abadi et al. [19]. It is also important to note that accounting for ε in stochastic gradient descent is still a loose upper bound and the actual upper bound is likely to be significantly lower. In more recent work, Beaulieu-Jones et al. [24] found that the Renyi

Differential privacy [25] calculates a tighter bound of privacy. For our use case the Renyi privacy cost was roughly one fourth the moments accountant used in this work. Renyi differential privacy could be used to reduce the epsilon value reported in this work.

Training AC-GANs in a Differentially Private Manner

We trained under differential privacy by limiting the effect any single SPRINT study participant has on the training process and by adding random noise based on the maximum effect of a single study participant. From the technical perspective, we limited the effect of participants by clipping the norm of the discriminator's training gradient and added proportionate Gaussian noise. This combination ensures that training cannot be tied to an individual and that it could have been guided by a different subject within or outside the real training data. The maximum effect of an outlier is limited and bounded. Comparing the neural network loss functions of the private and non-private training process demonstrates the effects of these constraints. Under normal training the losses of the generator and discriminator converged to an equilibrium before eventually increasing steadily (Supp. Fig. 1D). Under differentially private training the losses converged to and remained in a noisy equilibrium (Supp. Fig. 1F). At the beginning of training the neural networks changed rapidly. As training continued and the model achieved a better fit these steps, the gradient, decreased. Eventually the gradient becomes too small in comparison to the noise for training to continue any further.

As the models achieve better fit, the gradient shrinks, causing the gradient to noise ratio to decrease. This can occasionally lead to the private generator and discriminator falling out of sync (Supp. Fig. 3) or more commonly the private model generating less realistic samples due to

noise. To best select epochs, or training steps, where synthetic samples closely resemble real samples, we tested each epoch's data by training an additional classifier that must distinguish whether a generated participant was a part of the normal or intensive treatment groups.

During the training of AC-GAN, the only part that requires direct access to the private (real) data was the training of the discriminator. To achieve differential privacy, we only needed to "privatize" the training of the discriminators. The differential privacy guarantee of the entire AC-GAN directly followed because the output generative models are simply post-processing from the discriminator. We trained the discriminator using a differentially private version of the Adam method [26]. The standard Adam method iteratively updated the model parameters based on the gradients of the underlying loss function. To preserve privacy, we added noise to the gradient computed at each step as follows: first, we to ensured that the ℓ^2-norm of the gradient is bounded by clipping the gradient; then we perturbed each coordinate of the gradient by adding noise drawn from the Gaussian distribution with mean 0 and standard deviation proportional to the gradient clip size. The more noise we added (relative to the clipped norm of the gradient) the better the privacy guarantee we provide.

Due to the noisy training process, the losses of the discriminator and generator do not always converge (Supp. Fig. 1) and the training algorithm may have to be rerun. To properly account the total privacy loss from all the runs, we started with a target privacy budget (given by privacy parameters ε and δ) and repeatedly ran the private training algorithm until the AC-GAN converges or the privacy budget is exhausted. We used the moments accountant described in Abadi et al. [19] to keep track of the privacy parameters (ε, δ) over time.

We clipped the ℓ^2-norm of the gradient at 0.0001 and added noise from a normal distribution with a σ^2 of 1 ($\mathcal{N}(0, 1 * (0.0001^2))$). In our experiment, the AC-GAN trained in the second run of the algorithm converged, and the entire training process incurred a privacy loss within the budget ($\varepsilon = 4$, $\delta = 10^{-5}$).

Differentially Private Model Selection

We trained under differential privacy by limiting the effect any single SPRINT study participant has on the training process and by adding random noise based on the maximum effect of a single study participant. To do this, we first clipped the norm of the discriminator's training gradient. This clipping provides an upper bound on the maximum effect of a single study participant. We then added Gaussian noise according to this upper bound and the specified acceptable privacy loss. This combination ensures that training cannot be tied to an individual and that it could have been guided by a different subject within or outside the real training data. The maximum effect of an outlier is limited and bounded. Comparing the neural network loss functions of the private and non-private training process demonstrates the effects of these constraints. Under normal training the losses of the generator and discriminator converged to an equilibrium before eventually increasing steadily (Supp. Fig. 1D). Under differentially private training the losses converged to and remained in a noisy equilibrium (Supp. Fig. 1F). At the beginning of training the neural networks changed rapidly. As training continued and the model achieved a better fit these steps, the gradient, decreased. Eventually the gradient becomes too small in comparison to the noise for training to continue any further.

As the models achieve better fit, the gradient shrinks, causing the gradient to noise ratio to decrease. This can occasionally lead to the private generator and discriminator falling out of sync (Supp. Fig. 2) or more commonly the private model generating less realistic samples due to noise. To best select epochs, or training steps, where synthetic samples closely real samples, we tested each epoch's data by training an additional classifier that must distinguish whether a generated participant was a part of the normal or intensive treatment groups.

We found that sampling from multiple different epochs throughout training provided a more diverse training set. This provided summary statistics closer to the real data and higher accuracy in the transfer learning task. During the GAN training, we saved all the generative models across all epochs. We then generated a batch of synthetic data from each generative model and used a machine learning algorithm (logistic regression or random forest) to train a prediction model based on each synthetic batch of data. We then tested each prediction model on the training set from the real dataset and calculate the resulting accuracy. This testing is done only against the training set to ensure the test set is only used for evaluation. To select epochs that generate training data for the most accurate models under differential privacy, we used the standard "Report Noisy Min" subroutine: first add independent Laplace noise to the accuracy of each model (drawn from Lab($1/(n*\varepsilon)$)) to achieve (ε, 0) differential privacy where n is the size of the private dataset we perform the prediction on and output the model with the best noisy accuracy. Unlike the differentially private stochastic gradient descent training process, the "Report Noisy Min" subroutine can be performed with $\delta = 0$ [17]. We report the aggregate of these two processes to determine the upper bound of privacy risk - ($\varepsilon = 4.5$, $\delta = 10^{-5}$),

In practice, we choose the top five models that performed best on the transfer learning task for the training data using both logistic regression classification and random forest classification (for a total of 10 models). We performed this task under (0.5, 0)-differential privacy. In each of the ten rounds of selection, ε was set to 0.05. We found that in simulated cases of the “Report Noisy Min” subroutine this provided a strong signal to noise ratio (i.e. returned 10 values from the top 5% of epochs >99% of the time), while only adding a total of 0.5 ε. The ε value is a hyperparameter that could be optimized depending on the application.

Supplementary Online Results:

Supplemental Figure 1. AC-GAN architecture and training. A.) Structure of an AC-GAN. B.) The generator model takes a class label representing the treatment group (e.g. intensive or standard care group) and random noise as input and outputs a 3x12 vector for each participant (SBP, DBP and medication counts at each time point). C.) The discriminator model takes both real and simulated samples as input and learns to predict the source and a class label (i.e. normal or intensive treatment group). D.) Training loss for a non-private AC-GAN. E.) Training loss for a private AC-GAN.

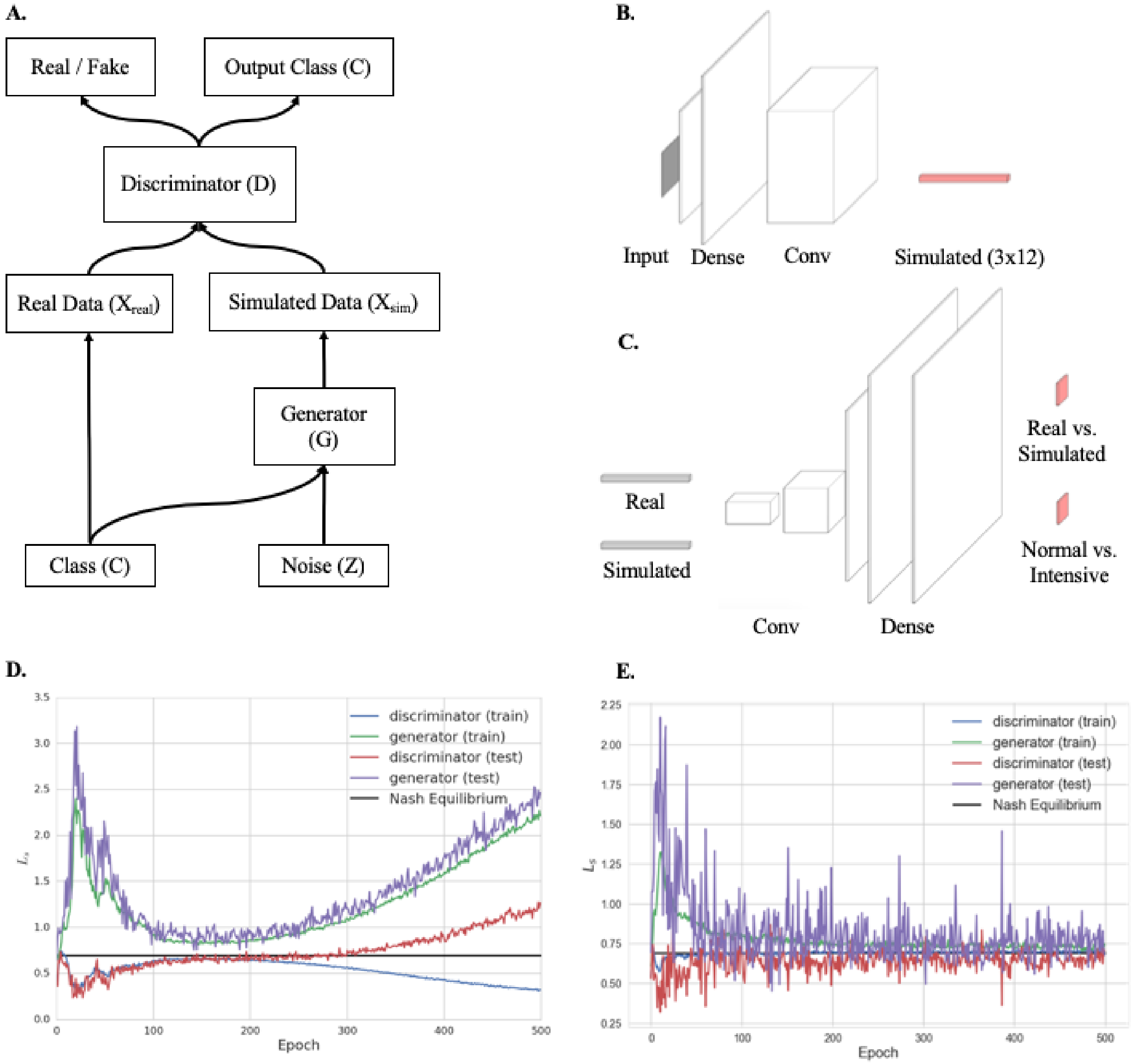

Supplemental Figure 2. Median Systolic Blood Pressure Trajectories from initial visit to 27 months. **A.)** Simulated samples (private and non-private) generated from the final (500th) epoch of training. **B.)** Simulated samples generated from the epoch with the best performing logistic regression classifier. **C.)** Simulated samples from the epoch with the best performing random

forest classifier. **D.)** Simulated samples from the top five random forest classifier epochs and top five logistic regression classifier epochs.

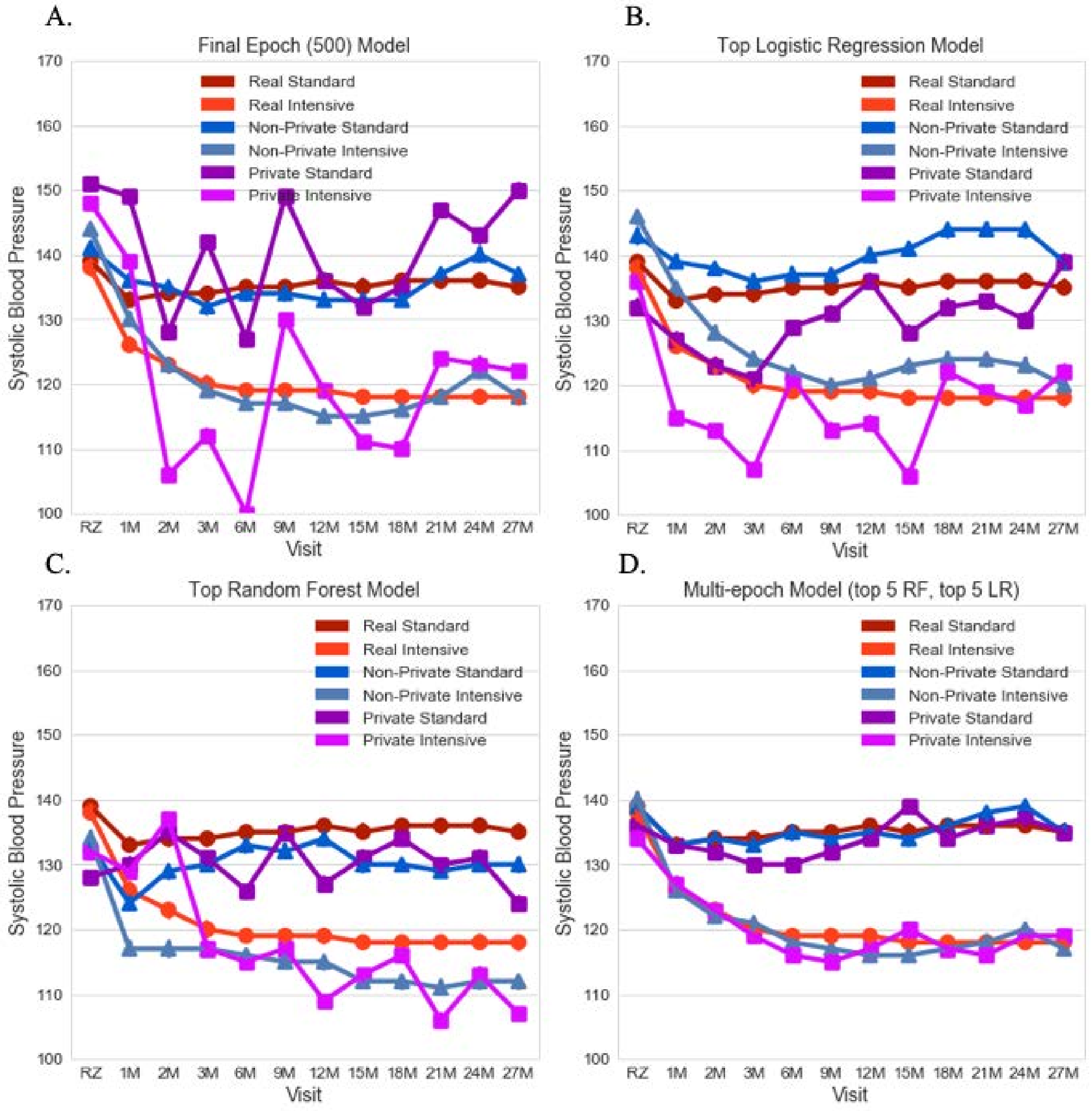

We applied two common machine learning classification algorithms and selected the top epochs in a differentially private manner (Supp. Fig. 2B and 2C). However, selecting only a single epoch does not account for the AC-GAN training process. Because the discriminator and generator compete from epoch to epoch, their results can cycle around the underlying distribution. The non-private models consistently improved throughout training (Supp. Fig. 4A, Supp. Fig. 5A), but this could be due to the generator eventually learning characteristics specific to individual participants. We observed that epoch selection based on the training data was important for the generation of realistic populations from models that incorporated differential privacy (Supp. Fig. 4B, Supp. Fig. 5B). To address this, we simulated 1,000 participants from

each of the top five epochs by both the logistic regression and random forest evaluation on the training data and combined them to form a multi-epoch training set. This process maintained differential privacy and resulted in a generated population that, throughout the trial, was consistent with the real population (Supp Fig. 2D). The epoch selection process was independent of the holdout testing data.

Supplemental Figure 3. Random noise breaks equilibrium.

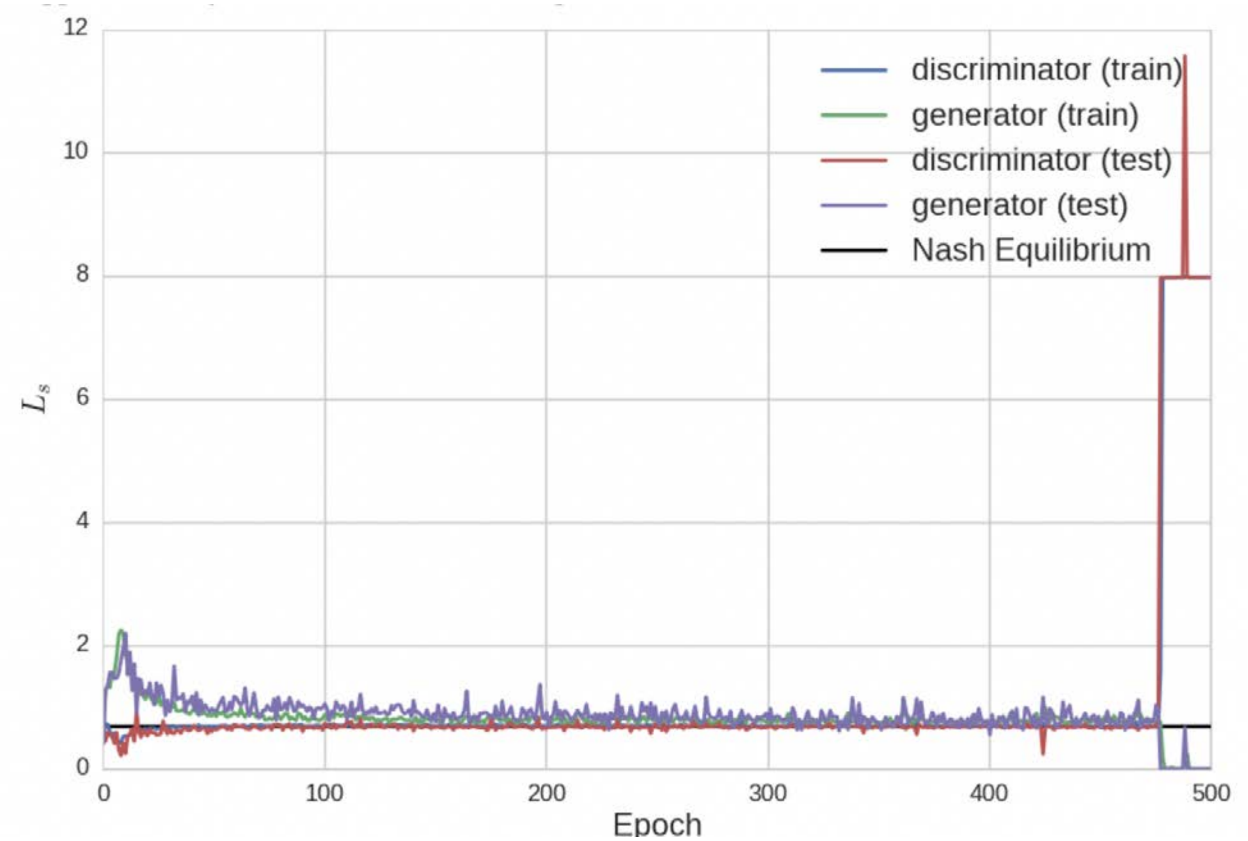

Supplemental Figure 4. Top Ranking Epochs for Transfer Learning Exercise.

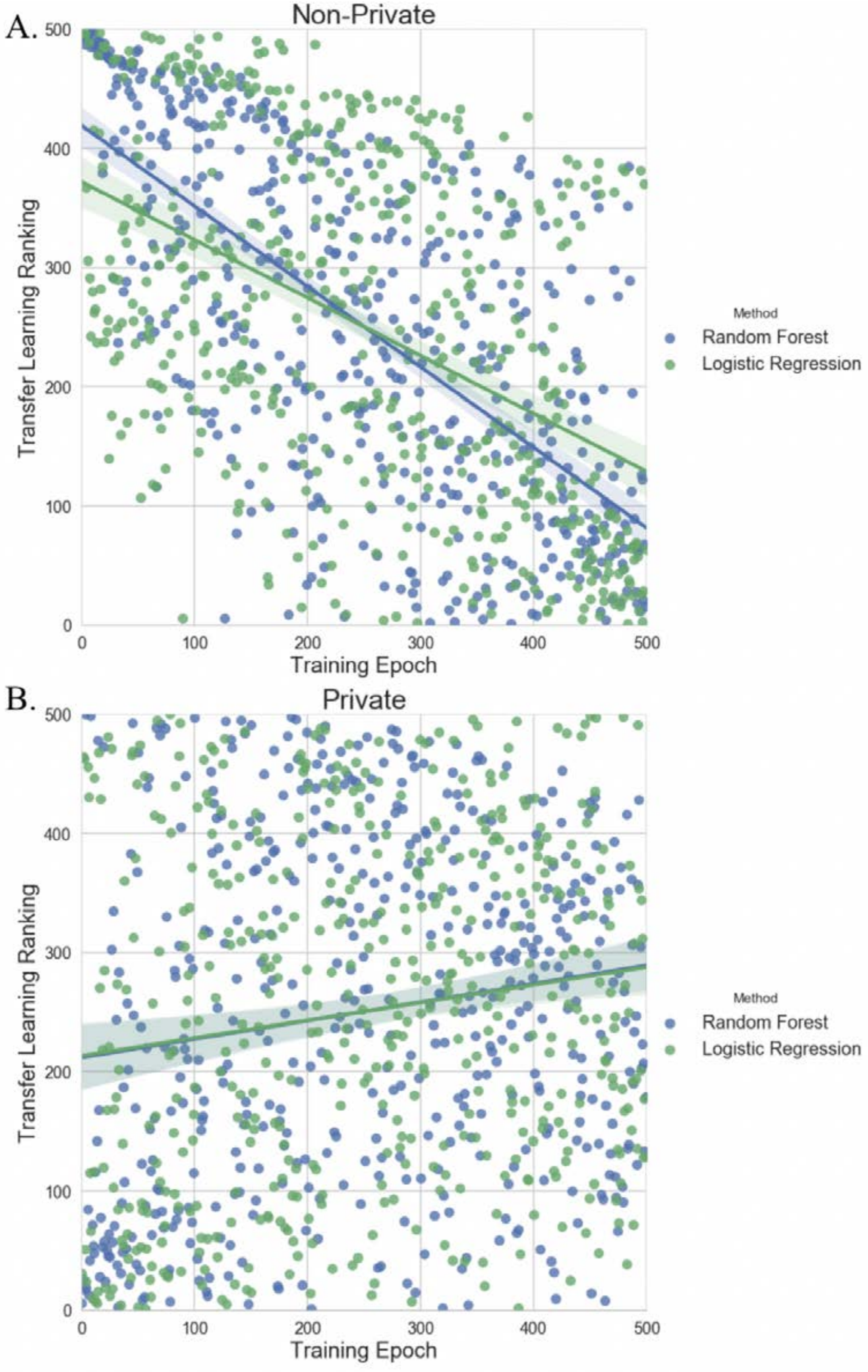

Supplemental Figure 5. Scores vs. Epoch for Transfer Learning Task.

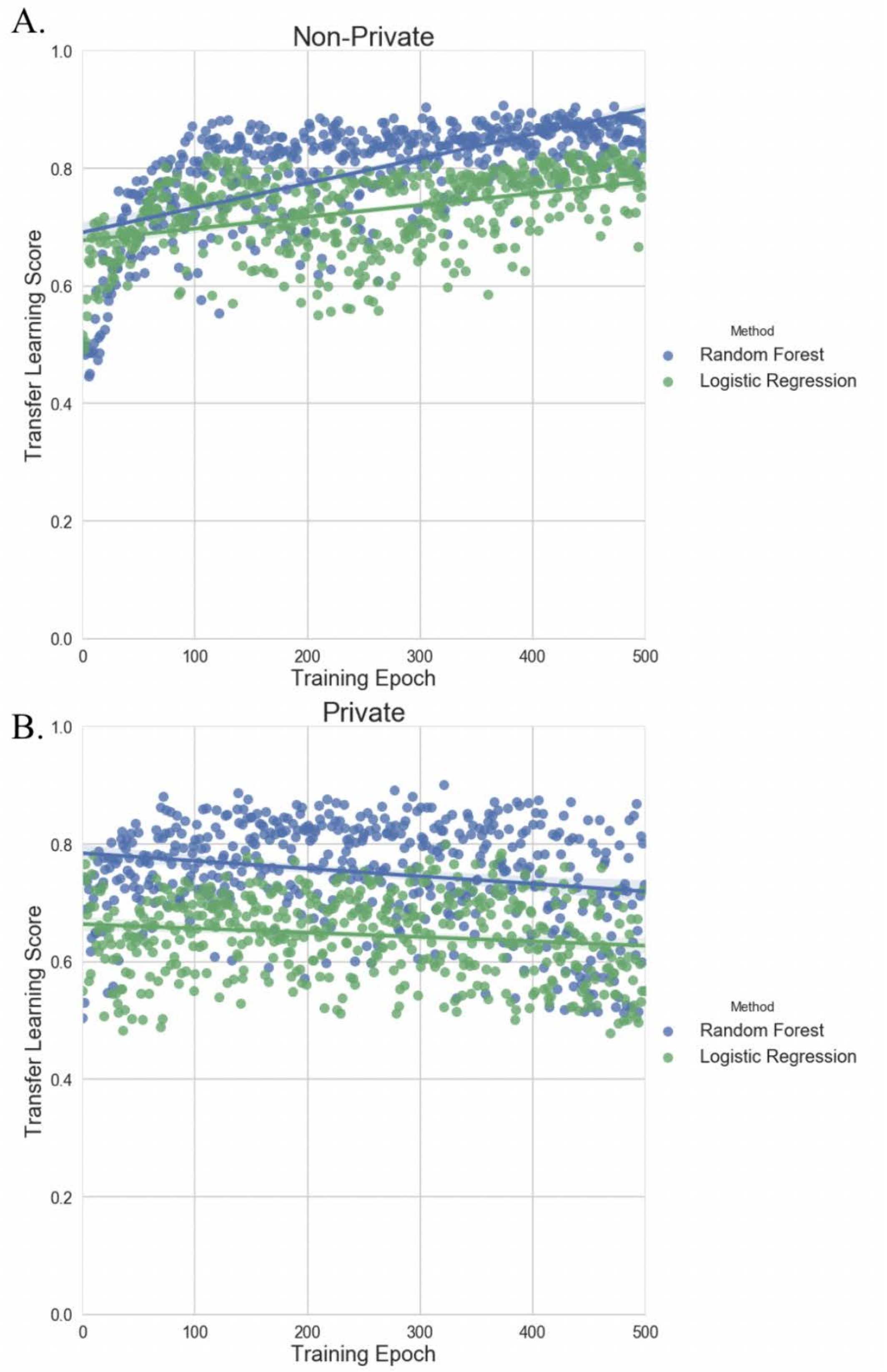

Supplemental Figure 6. A.) Random forest variable importance scores by training data. **B.)** Logistic Regression variable coefficients by training data. **C.)** Support Vector Machine variable coefficients by training data.

A.

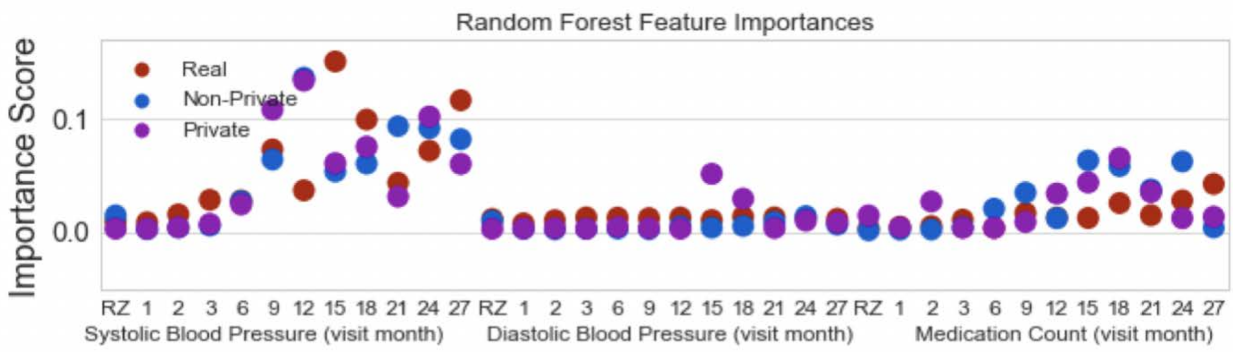

B.

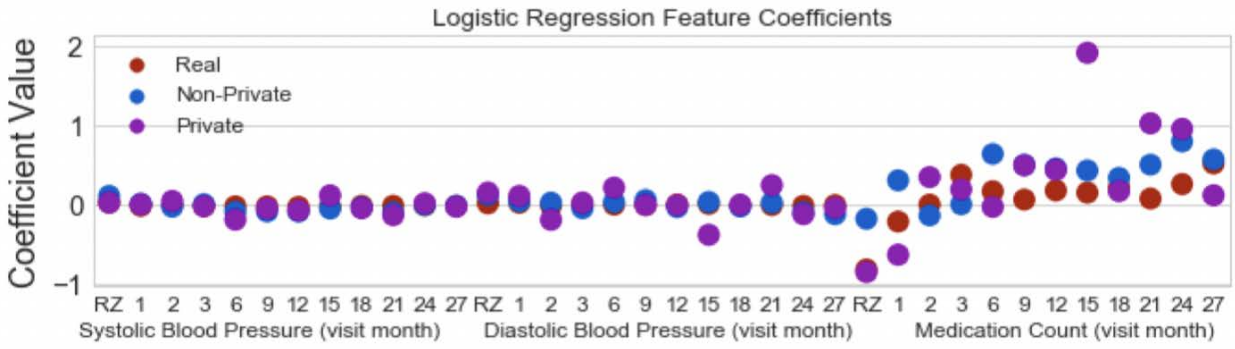

C.

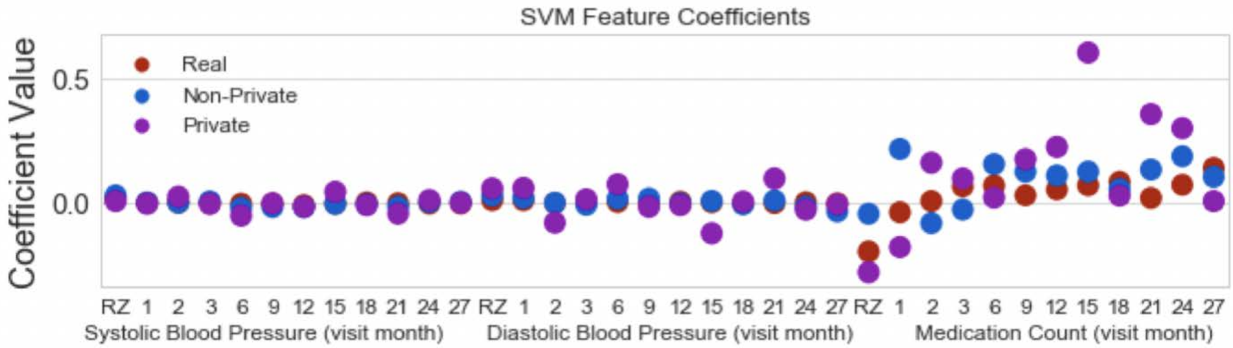

Supplemental Figure 7. Feature correlation between cross-validation for the real data.

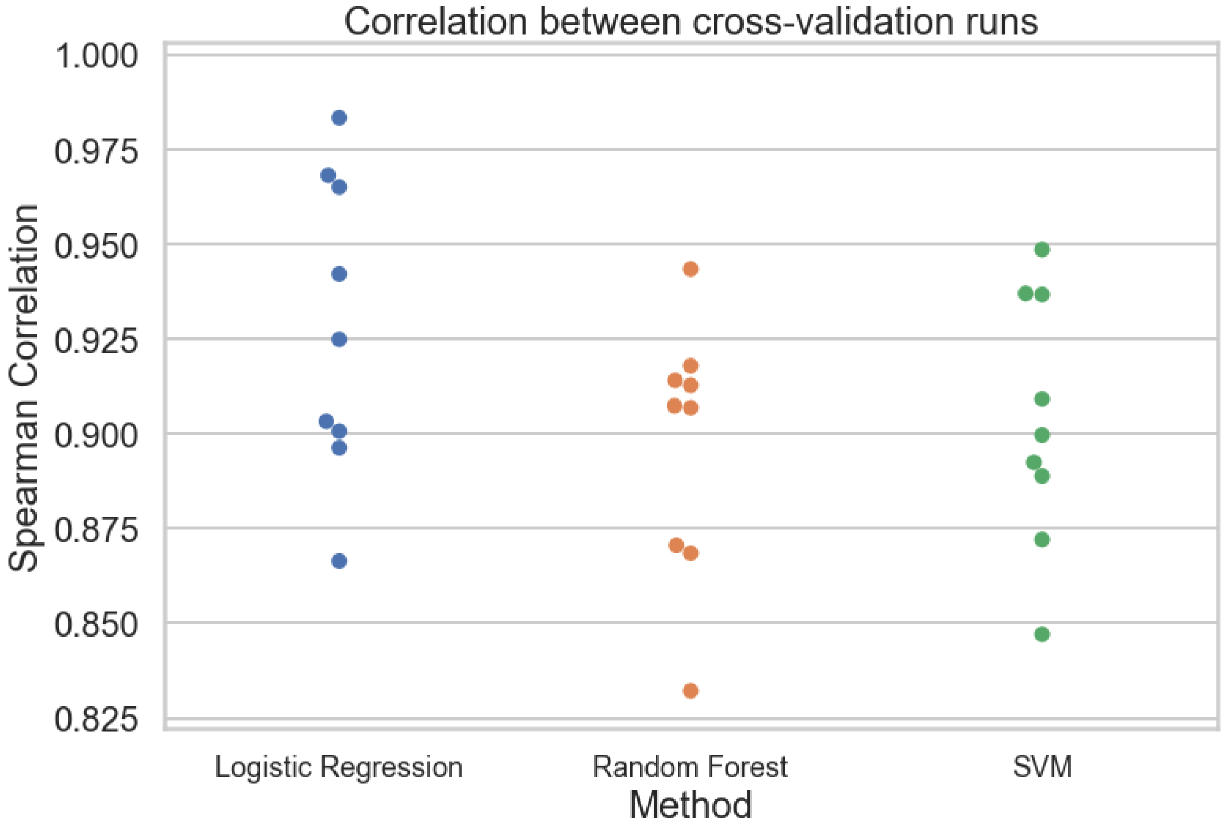

Supplemental Table 1. Spearman Correlation between variable importance scores (Random Forests) and model coefficients (Support Vector Machine and Logistic Regression) for the SPRINT trial data.

	**Random Forest**		**Support Vector Machine**		**Logistic Regression**	
	Correlation	P-Value	Correlation	P-Value	Correlation	P-Value
Real - Non-Private	0.7207	7.1518e-07	0.5279	9.35794e-04	0.6973	2.2950e-06
Real - Private	0.6123	7.21008e-05	0.6517	1.6644e-05	0.6893	3.3310e-06

Supplemental Table 2. Spearman Correlation between variable importance scores (Random Forests) and model coefficients (Support Vector Machine and Logistic Regression) for the MIMIC critical care data.

	**Random Forest**		**Support Vector Machine**		**Logistic Regression**	
	Correlation	P-Value	Correlation	P-Value	Correlation	P-Value
Real - Non-Private	0.4059	0.00566	0.2952	0.04894	0.4268	0.00345

Supplemental References

[1] Privacy-preserving generative deep neural networks support clinical data sharing, Github Repository. Doi: 10.5281/zenodo.2647635. Available at: https://github.com/greenelab/SPRINT_gan.

[2] Beaulieu-Jones BK, Greene CS. Reproducibility of computational workflows is automated using continuous analysis. Nat Biotechnol 2017;35. doi:10.1038/nbt.3780.

[3] Beaulieu-Jones, Brett (2017): SPRINT Private GAN Docker Image. Doi: 10.6084/m9.figshare.5165731.v1 Available at: https://figshare.com/articles/SPRINT_Private_GAN_Docker_Image/5165731/1

[4] Choi, E., Biswal, S., Malin, B., Duke, J., Stewart, W.F. and Sun, J., Generating Multilabel Discrete Electronic Health Records Using Generative Adversarial Networks. arXiv.org Available at: https://arxiv.org/abs/1703.06490. Accessed April 19, 2019.

[5] Esteban, C., Hyland, S.L. and Rätsch, G., 2017. Real-valued (medical) time series generation with recurrent conditional gans. arXiv.org Available at: https://arxiv.org/abs/1706.02633. Accessed April 19, 2019.

[6] Garfinkel, S.L., 2015. De-identification of personal information. NISTIR, 8053, pp.1-46.

[7] El Emam K, Jonker E, Arbuckle L, Malin B, Riedl J. A Systematic Review of Re-Identification Attacks on Health Data. PLoS One 2011;6:e28071.

[8] Malin B, Sweeney L. How (not) to protect genomic data privacy in a distributed network: using trail re-identification to evaluate and design anonymity protection systems. J Biomed Inform 2004;37:179–192.

[9] Kohane IS, Altman RB. Health-Information Altruists? A Potentially Critical Resource. N Engl J Med 2005;353:2074–2077.

[10] Sweeney, L., 2002. k-anonymity: A model for protecting privacy. International Journal of Uncertainty, Fuzziness and Knowledge-Based Systems, 10(05), pp.557-570.

[11] Sweeney, L., Abu, A. and Winn, J., 2013. Identifying participants in the personal genome project by name (a re-identification experiment). arXiv.org Available at: https://arxiv.org/abs/1304.7605. Accessed April 19, 2019.

[12] Narayanan, A. and Shmatikov, V., 2008. Robust de-anonymization of large datasets (how to break anonymity of the Netflix prize dataset). Proceedings of the 2008 IEEE Symposium on Security and Privacy (pp. 111-125).

[13] Homer, N., Szelinger, S., Redman, M., Duggan, D., Tembe, W., Muehling, J., Pearson, J.V., Stephan, D.A., Nelson, S.F. and Craig, D.W., 2008. Resolving individuals contributing trace amounts of DNA to highly complex mixtures using high-density SNP genotyping microarrays. PLoS genetics, 4(8), p.e1000167.

[14] Fredrikson, M., Jha, S. and Ristenpart, T., 2015, October. Model inversion attacks that exploit confidence information and basic countermeasures. Proceedings of the 22nd ACM SIGSAC Conference on Computer and Communications Security (pp. 1322-1333).

[15] Shokri, R., Stronati, M., Song, C. and Shmatikov, V., 2017, May. Membership inference attacks against machine learning models. 2017 IEEE Symposium on Security and Privacy (SP) (pp. 3-18).

[16] Fredrikson, M., Lantz, E., Jha, S., Lin, S., Page, D. and Ristenpart, T., 2014. Privacy in pharmacogenetics: An end-to-end case study of personalized warfarin dosing. 23rd {USENIX} Security Symposium ({USENIX} Security 14) (pp. 17-32).

[17] Dwork, C. and Roth, A., 2014. The algorithmic foundations of differential privacy. Foundations and Trends® in Theoretical Computer Science, 9(3–4), pp.211-407.

[18] Simmons, S. and Berger, B., 2016. Realizing privacy preserving genome-wide association studies. Bioinformatics, 32(9), pp.1293-1300.

[19] Abadi, M., Chu, A., Goodfellow, I., McMahan, H.B., Mironov, I., Talwar, K. and Zhang, L., 2016, October. Deep learning with differential privacy. Proceedings of the 2016 ACM SIGSAC Conference on Computer and Communications Security (pp. 308-318). ACM.

[20] Shokri, R. and Shmatikov, V., 2015, October. Privacy-preserving deep learning. Proceedings of the 22nd ACM SIGSAC conference on computer and communications security (pp. 1310-1321).

[21] Odena, A., Olah, C. and Shlens, J., 2017, August. Conditional image synthesis with auxiliary classifier gans. Proceedings of the 34th International Conference on Machine Learning-Volume 70 (pp. 2642-2651).

[22] Chollet, F., 2015. Keras.

[23] Dwork, C., McSherry, F., Nissim, K. and Smith, A., 2006, March. Calibrating noise to sensitivity in private data analysis. Theory of cryptography conference (pp. 265-284). Springer, Berlin, Heidelberg.

[24] Beaulieu-Jones, Brett K., William Yuan, Samuel G. Finlayson, and Zhiwei Steven Wu. "Privacy-Preserving Distributed Deep Learning for Clinical Data." NeurIPS Machine Learning for Health Workshop arXiv:1812.01484 (2018).

[25] Mironov, I., 2017, August. Rényi differential privacy. 2017 IEEE 30th Computer Security Foundations Symposium (CSF) (pp. 263-275).
[26] Kingma, D.P. and Ba, J., 2014. Adam: A method for stochastic optimization. arXiv.org Available at: https://arxiv.org/abs/1412.6980. Accessed April 19, 2019.
